# Clinical Evaluation of Acute Pancreatitis Caused by SARS-CoV-2 Virus Infection

**DOI:** 10.1155/2021/5579795

**Published:** 2021-05-03

**Authors:** Hulya Vatansev, Mehmet Aykut Yıldırım, Serkan Kuccukturk, Mehmet Ali Karaselek, Cengiz Kadiyoran

**Affiliations:** ^1^Pulmonary Diseases Department, Meram Faculty of Medicine, Necmettin Erbakan University, Konya, Turkey; ^2^Department of General Surgery, Meram Medicine Faculty, Necmettin Erbakan University, Konya, Turkey; ^3^Department of Medical Biology, Meram Faculty of Medicine, Necmettin Erbakan University, Konya, Turkey; ^4^Department of Hematology, Meram Faculty of Medicine, Necmettin Erbakan University, Konya, Turkey; ^5^Radiology Department, Meram Faculty of Medicine, Necmettin Erbakan University, Konya, Turkey

## Abstract

**Introduction:**

Coronavirus 2019 disease (COVID-19), caused by the severe acute respiratory syndrome coronavirus-2 (SARS-CoV-2), has spread to more than 200 countries worldwide. We aimed to present acute pancreatitis (AP) cases caused by SARS-CoV-2 viral infection.

**Methods:**

The study was conducted retrospectively between April 2020 and June 2020 in Necmettin Erbakan University Meram, Medical Faculty Hospital, and 150 hospitalized patients diagnosed with COVID-19 were included. The degree of acute pancreatitis was determined according to the Atlanta classification. Organ failures of the patients were evaluated in terms of respiratory, cardiovascular, and nephrology according to the modified Marshall scoring (MMS) system, and CTSI (Balthazar score) and Imrie score were determined. Modified Marshall score ≥ 2 was considered organ failure.

**Results:**

A total of 29 patients were diagnosed with acute pancreatitis. All 29 patients with pancreatitis had respiratory failure during hospitalization. After the diagnosis of pancreatitis, there was no change in respiratory failure. According to the Atlanta classification, 19 patients had mild AP and 10 patients had moderate AP. Patients with acute pancreatitis were scored according to the CTSI (Balthazar score), and there were no patients with ≥6 severe pancreatitis. The CTSI score of 4 patients was 3. In addition, the Imrie score of the patients was determined and 8 patients with Imrie score ≥ 3 were identified.

**Conclusion:**

The rate of pancreatic damage in SARS-CoV-2 infection was found to be 19% (*n* = 29) in our study. In our study, we highlight acute pancreatitis as a complication associated with COVID-19 and the importance of pancreatic evaluation in patients with COVID-19 and abdominal pain is demonstrated.

## 1. Introduction

The world experienced two global pandemics: severe acute respiratory failure syndrome (SARS) in 2002-2003 and the Middle East respiratory syndrome (MERS) in 2011. The reason for both outbreaks was identified as zoonotic origin betacoronavirus (CoV). At the end of 2019, “Corona Virus 2019 Disease (COVID-19)” caused by a new type of betacoronavirus named SARS-CoV-2 in Wuhan city of China emerged as a new pandemic [1]. To date, approximately 24.8 million COVID-19 cases have been identified worldwide and around 838,924 deaths have been reported [2].

The SARS-CoV-2 virus is transmitted by inhalation and uses the angiotensin-converting enzyme 2 (ACE2) receptor to enter the cell. ACE2 is expressed in alveolar epithelial cells as well as in the heart, gastrointestinal tract, kidney, testicles, and other organs. This means that SARS-CoV-2 enters other tissues and organs through ACE2 and causes multiple organ damage [3, 4].

In recent studies, SARS-CoV-2 was found to cause heart, kidney, liver damage, and gastrointestinal symptoms, in addition to lung disorders such as ARDS [5]. Symptoms of the virus include fever, cough, sore throat, shortness of breath, fatigue, and weakness [6–8]. In addition, gastrointestinal symptoms such as nausea, vomiting, diarrhea, and abdominal pain may occur in COVID-19 [9, 10]. Few cases of COVID-19 acute pancreatitis (AP) were reported in the literature [11, 12]. In the light of clinical and literature data, pancreatic damage may occur in some COVID-19 patients. During the clinical follow-up of COVID-19 patients, we noticed that some patients with widespread abdominal pain had pancreatic damage along with elevated serum amylase and lipase. This study is aimed at investigating the association of acute pancreatitis with COVID-19.

## 2. Methods

The study was conducted retrospectively between April 2020 and June 2020 in Necmettin Erbakan University, Meram Medical Faculty Hospital, and 150 hospitalized patients diagnosed with COVID-19 were included. This study was approved by the Institutional Review Board at Necmettin Erbakan University, Meram Medical Faculty (protocol number: 2020/2469). Patients with acute onset, extensive abdominal pain, increased serum amylase and lipase, and findings of acute pancreatitis on intravenous contrast-enhanced abdominal tomography were included in the study. Patients with gallstones, hypercalcemia, hypertriglyceridemia, alcohol use history, and chronic pancreatitis were excluded from the study. Complete blood count, glucose, urea, creatinine, BUN, albumin, AST, ALT, LDH, lactate, C-reactive protein (CRP), procalcitonin (PCT), calcium (Ca), and arterial blood gas values were measured for the patients. The degree of acute pancreatitis was determined according to the Atlanta classification [13]. Organ failures of the patients were evaluated in terms of respiratory, cardiovascular, and nephrology systems according to the modified Marshall scoring (MMS) system [14, 15], and CTSI (Balthazar score) and Imrie score were determined. Modified Marshall score ≥ 2 was considered organ failure. Accordingly, patients with no local complications and organ failure within the first 48 hours were graded as mild; those with local complications or organ failure in the first 48 hours were graded as moderate, and patients with permanent organ failure affecting one or more organs after 48 hours were graded as severe. SARS-CoV-2 positivity of the patients was detected with the real-time transcription-polymerase chain reaction method. Lung CT images of COVID-19 were evaluated and divided into 4 groups as negative, atypical, indeterminate, and typical [16]. After all evaluations, the association of COVID-19 diagnosis with acute pancreatitis was examined in detail. Respiratory and gastrointestinal symptoms of patients diagnosed with COVID-19 were monitored daily and recorded.

Anticoagulant, hydroxychloroquine, favipiravir, antibiotics, and liquid electrolyte replacement therapy were used for the treatment of patients diagnosed with COVID-19 according to clinical and laboratory findings. Oxygen support was provided with nasal cannula or high-flow in patients with low oxygen saturation in the blood [17]. Symptomatic treatment was applied to patients who showed signs of AP.

Patients who were desaturated despite oxygen support and those who were found to have multiorgan failure and who were hemodynamically unstable were followed up in the intensive care unit. According to the Atlanta classification, the patients in our study did not have severe pancreatitis, and therefore, nutrition was performed enterally in all patients. The data for all patients from hospitalization to discharge were recorded in the study file.

## 3. Results

**Figure 1:**
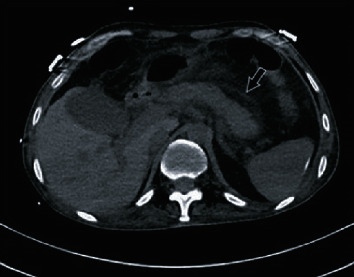
Abdominal noncontrast-enhanced computed tomography in a 44-year-old man recovering from severe coronavirus disease 2019 pneumonia with signs of acute pancreatitis, including inflammatory changes in peripancreatic fat in the pancreas (white-framed arrow).

The study included 29 patients with COVID-19 diagnosis who developed AP. M : F ratio was 18/11, and mean age was 62.22 ± 16.37 and 67.09 ± 19.02, respectively. Laboratory findings of patients with AP are shown in Table 1. According to interpretation of lung CT images, 2 patients were negative, 11 patients were atypical, 7 patients were uncertain, and 9 patients were typical. During the follow-up of the patients, signs of pancreatitis were detected in 29 patients (Figure 1). These 29 patients were evaluated in terms of organ failure according to MMS, and serious organ failure was detected in 3 patients. According to the Atlanta classification, 19 patients had mild AP and 10 patients had moderate AP. Patients with acute pancreatitis were scored according to the CTSI (Balthazar score) and there were no patients with ≥6 severe pancreatitis. The CTSI score of 4 patients was 3. In addition, the Imrie score of the patients was determined and 8 patients with Imrie score ≥ 3 were identified (Table 2). All 29 patients with pancreatitis had respiratory failure during hospitalization. After the diagnosis of pancreatitis, there was no change in respiratory failure.

Serum amylase/lipase levels of all patients with AP symptoms gradually decreased after an average of 5 days. Nausea and vomiting were more pronounced in patients with AP than in other patients. Intravenous chlorpropamide and ondansetron were used in these patients until the symptoms were controlled. During the first 5 days, 10 patients were fed parentally due to intense nausea and vomiting. In 19 patients, nausea-vomiting was controlled with antiemetic treatment. In spite of dominant enteral feeding, these patients were supported with intravenous hydration. Abdominal examination of the other patients was normal, and gastrointestinal system complaints were not detected in the patients. There were no or less complaints of nausea and vomiting in the other patients during follow-up, except for the 29 patients with AP. Enteral nutrition was given to all patients who could tolerate oral nutrition.

While 72% of 29 patients were discharged after healing, 28% of patients died due to respiratory failure and multiorgan failure. While the hospitalization period of patients with AP was 12 (2-30) days on average, the duration of hospitalization of patients without AP was determined as 4 days [1–13]. While mortality was 8% in patients without AP, it was determined as 28% in patients with AP. AP status in COVID-19 affects hospital stay and mortality rate.

When patients without pancreatic damage and patients with AP were compared in terms of laboratory parameters, CRP, procalcitonin, D-dimer, ferritin, glucose, creatine, lactate, and LDH were found to be high, and lymphocyte count was low. In addition, kidney damage (abnormal creatinine) was detected in 12 out of 29 patients with AP.

## 4. Discussion

SARS-CoV-2 infection, which started in China and spread around the world, is a very serious public health problem [5]. Lack of specific drugs effective against the disease and the presence of asymptomatic infected individuals increase the spread of the disease [18]. Patients infected with SARS-CoV-2 mainly suffer from systemic symptoms such as fever and fatigue, respiratory symptoms such as cough and sputum production, and digestive symptoms such as diarrhea [6]. Using the ACE2 receptor to enter the cells, SARS-CoV-2 causes more damage to tissues expressing this receptor. Although the lung is considered to be the major expressing organ of this receptor, after the detection of many organs besides the lung which express ACE2, it was considered that serious damage would occur in other organs. One of these organs is the pancreas. ACE2 is highly expressed in pancreatic islets, and SARS-CoV-2 infection was reported to cause damage to the islets followed by acute diabetes [19]. Of our 29 COVID-19 patients with pancreatic damage, 15 of them had abnormal blood glucose. These findings suggest that pancreatic damage in COVID-19 may be directly caused by the cytopathic effect mediated by SARS-CoV-2 replication. Although the disease is mild in most patients with AP, which is an inflammatory disease of the pancreas, it can also be seen as a severe, complicated disease accompanied by necrosis at a rate of 15-25% and organs such as the heart, liver, and kidneys are affected. In parallel with this information, kidney damage (abnormal creatinine) was detected in addition to pancreatic damage in 12 patients in our study.

In the 29 patients we followed in our study, no patient had severe pancreatitis. Despite all the advances in technology, it is a disease with high morbidity and mortality [20, 21]. In acute pancreatitis, pathological findings can be seen in a wide range from mild interstitial edema to severe hemorrhagic gangrene and necrosis. Likewise, clinical symptoms may occur to varying degrees, ranging from abdominal pain to hypotension, fluid sequestration, metabolic disorders, sepsis, septic shock, and multiorgan failure [21, 22]. Acute pancreatitis may be due to many etiological reasons such as gallstones, alcohol use, hyperlipidemia, biliary strictures, post-ERCP, genetic factors, pancreatic duct injury, drug use, and hypercalcemia. In the literature, it was reported that pancreatitis may develop in viral infections such as coxsackievirus, HIV, hepatitis b cytomegalovirus, varicella-zoster, and herpes simplex [23, 24]. It was also shown in previous SARS-CoV-2 infections that the virus can damage not only tissues such as the lung, liver, and kidney, but also the pancreas [25]. Pancreatic damage due to COVID-19 infection was reported at 17%. Among the AP findings of these patients, mild amylase and lipase elevation were found [19]. The findings in our patients were compatible with the literature. Although the exact pathogenesis of this damage is not known, it is thought to be mediated by ACE2, which is densely found in pancreatic tissue [4]. In our study, detection of acute pancreatitis case with pancreatic damage in 19% of 150 patients diagnosed with COVID-19 is consistent with the literature.

In COVID-19 disease, especially in serious patients, patients should be treated in hospital with inpatient supportive treatments. The length of stay is prolonged in cases where the SARS-CoV-2 virus affects more organs. In our study, the average length of stay in patients without AP was found to be 3.76 days, while it was found to be 12 days for patients with AP, and the AP status increased the duration of hospital stay by approximately 4 times. Accordingly, it was found that mortality rates are increased compared to patients without AP.

Our study has some limitations. We observed AP as a clinical outcome in COVID-19 patients. Isolation of the virus from pancreas tissue was not performed. The ACE2 receptors found in excess in pancreas tissue are pathophysiologically thought to lead to overexpression of SARS-CoV-2. Additionally, with the severe pulmonary involvement in some patients, it is not fully possible to say whether ARDS is a primary effect linked to SARS-CoV-2 or a secondary effect linked to AP. However, it may be considered that AP aggravates pulmonary failure that is present.

## 5. Conclusion

In this study, during our clinical observations, patients with COVID-19, in whom we detected elevated serum amylase and lipase and AP findings in CT along with abdominal pain, attracted our attention. Based on this point, it was shown that there may be pancreatic damage in SARS-CoV-2 infection, and this rate was found to be 19% in our study. These results suggest that there may be potential for mild pancreatic damage in patients with COVID-19 pneumonia. This may be associated with secondary enzyme abnormalities without direct viral involvement of the pancreas or significant pancreatic damage in the context of severe disease. As a result of the data obtained from our study, the hospitalization periods of patients with pancreatic damage are prolonged, mortality increased, and the treatment of COVID-19 is seriously affected.

As a result, it should be kept in mind that patients diagnosed with COVID-19 and who have abdominal pain may develop AP. The hospitalization duration lengthens, and mortality rates increase for COVID-19 patients developing AP.

## Figures and Tables

**Table 1:** Laboratory findings of patients with COVID-19 and acute pancreatitis.

Laboratory parameters (*N* = 29)	Mean	SEM
Age (year)	64.07	3.23
Duration of stay (day)	12.07	1.24
Initial HCT (%)	39.28	1.10
Initial HGB (g/dL)	12.67	0.38
Albumin (mg/dL)	34.90	1.49
Amylase (U/L)	132.28	26.16
CRP (mg/L)	62.42	10.73
D-dimer (quantitative)	839.45	270.57
Glucose (mg/dL)	127.09	8.79
HCT (%)	35.72	1.46
HGB (g/dL)	11.49	0.50
Calcium (mg/dL)	8.55	0.15
Creatinine (mg/dL)	1.50	0.34
Lactate	1.66	0.12
LDH (U/L)	331.28	56.36
Lipase (U/L)	116.64	33.36
mPO_2_ (%)	53.48	4.29
Procalcitonin	1.27	0.92
AST (U/L)	34.35	5.98
ALT (U/L)	26.22	4.98
Urea (mg/dL)	67.58	12.01
BUN	30.42	5.55
WBC (10^3^/Ul)	10.24	0.86

SEM: standard error mean; HCT: hematocrit; HGB: hemoglobin; CRP: C-reactive protein; ALT: alanineaminotransferase; AST: aspartate aminotransferase; LDH: lactate dehydrogenase; BUN: blood urea nitrogen.

**Table 2:** Lung CT and clinical scores of patients with COVID-19 and acute pancreatitis.

*N* = 29	Score	*n*	%
Lung CT	1	2	6.90
	2	11	37.93
	3	7	24.14
	4	9	31.03
Balthazar CT	1	16	55.17
	2	9	31.03
	3	4	13.79
MMS	0	10	34.48
	1	9	31.03
	2	7	24.14
	3	3	10.34
Atlanta	HAP	19	65.52
	MAP	10	34.48
Imrie	0	2	6.90
	1	9	31.03
	2	10	34.48
	3	6	20.69
	4	2	6.90

## Data Availability

The data used to support the findings of this study are available from the corresponding author upon request.
